# A One-Dimensional Depthwise Separable Convolutional Neural Network for Bearing Fault Diagnosis Implemented on FPGA

**DOI:** 10.3390/s24237831

**Published:** 2024-12-07

**Authors:** Yu-Pei Liang, Hao Chen, Ching-Che Chung

**Affiliations:** Department of Computer Science and Information Engineering, Advanced Institute of Manufacturing with High-Tech Innovations, National Chung Cheng University, Chia-Yi 621301, Taiwan; ypliang@cs.ccu.edu.tw (Y.-P.L.); eeericpig123@alum.ccu.edu.tw (H.C.)

**Keywords:** current signal fault diagnosis, depthwise separable convolution (DSC), neural networks, quantization, fixed-point arithmetic, real-time systems, digital circuits

## Abstract

This paper presents a hardware implementation of a one-dimensional convolutional neural network using depthwise separable convolution (DSC) on the VC707 FPGA development board. The design processes the one-dimensional rolling bearing current signal dataset provided by Paderborn University (PU), employing minimal preprocessing to maximize the comprehensiveness of feature extraction. To address the high parameter demands commonly associated with convolutional neural networks (CNNs), the model incorporates DSC, significantly reducing computational complexity and parameter load. Additionally, the DoReFa-Net quantization method is applied to compress network parameters and activation function outputs, thereby minimizing memory usage. The quantized DSC model requires approximately 22 KB of storage and performs 1,203,128 floating-point operations in total. The implementation achieves a power consumption of 527 mW at a clock frequency of 50 MHz, while delivering a fault diagnosis accuracy of 96.12%.

## 1. Introduction

With the advent of advanced industrialization, factory production has largely transitioned from manual labor to automation, with machines undertaking the majority of tasks. This shift has led to the development of industrial equipment that is increasingly complex, sophisticated, and intelligent. Consequently, greater attention is being directed toward ensuring the quality and reliability of the mechanical components that underpin these systems.

Rolling bearings are critical components in modern industrial machinery, playing a fundamental role in supporting rotating elements under load and directly influencing the performance and stability of the entire system [1]. Operating under the harsh conditions typical of industrial environments, bearings are subject to vibrations that can lead to aging, wear, and fatigue. Such degradation may result in bearing failures, potentially causing significant malfunctions, accidents, and economic losses. Research indicates that approximately 30% of failures in rotating machinery stem from bearing damage [2]. To mitigate unexpected downtime and associated costs, the development of advanced fault diagnosis techniques for rolling bearings has become a focal point of study [3].

Traditional data-driven methods for bearing fault diagnosis include model-driven, signal-driven, and knowledge-driven approaches, which rely on manually extracting features from data based on accumulated expertise. These features are subsequently used to train machine learning algorithms, such as support vector machines or random forests, to classify the health status of CNC machines. Techniques like fast Fourier transform (FFT) and wavelet transform are employed to reduce data dimensionality and enhance the efficiency of fault diagnosis by isolating key signal characteristics.

Zhang [4] proposed a feature extraction method for analog circuit incipient fault diagnosis that combines wavelet transform, Kernel Entropy Component Analysis (KECA), and One-Against-One Least Squares Support Vector Machine (OAO LSSVM), achieving significant improvements in diagnostic accuracy and dimensionality reduction. Subsequently, they introduced an enhanced wavelet transform approach [5] incorporating optimal wavelet basis function selection and a Multiple Kernel Extreme Learning Machine (MKELM) optimized using particle swarm, further advancing feature extraction and fault identification capabilities. On the other hand, researchers have developed diagnostic methodologies such as a fault diagnosis method leveraging Manhattan distance evaluation and voltage difference analysis [6], which provides sensitive and reliable detection and isolation of multiple faults. Additionally, Du [7] proposed an Integrated Gradient-based Continuous Wavelet Transform (IG-CWT) method to isolate critical frequency components for bearing fault diagnosis, enhancing data preprocessing for deep learning applications.

Data-driven methods, while effective, often demand substantial memory and computational resources for feature extraction, limiting their feasibility for edge computing applications. Similarly, statistical approaches face challenges in real-time applications due to their reliance on extensive pre-processing and batch computations. For instance, Tan [3] explored bearing fault diagnosis in rotating machinery using data from three accelerometers under normal operating conditions and three fault types (inner race defect, outer race defect, and ball defect). Statistical analysis of the sensor data extracted 14 features per sensor, yielding 42 features per rotation as input for a Deep Belief Network (DBN) trained for fault classification. However, calculating features such as skewness and kurtosis required storing 500 data points per sensor, making the approach impractical for hardware-based implementations. This limitation underscores its suitability for software-based processing rather than real-time health monitoring of CNC machine tools.

In summary, directly classifying real-time data offers a practical solution for field applications by eliminating the need for extensive preprocessing and large memory storage, thereby enhancing compatibility with real-time monitoring systems. While numerous studies have successfully employed neural network-based methods with various architectures [8,9,10,11,12,13,14,15,16,17] to achieve high diagnostic accuracy, these approaches often demand significant memory and hardware resources, making them unsuitable for devices with constrained hardware capabilities. This paper addresses these challenges by utilizing a depthwise separable convolution (DSC) to reduce the parameter count and computational complexity of convolution operations, thereby optimizing hardware resource usage. The study evaluates the proposed method using the Paderborn University (PU)-bearing dataset [18], a public dataset for bearing fault diagnosis that includes synchronized current and vibration signal data.

The main contributions in this paper are summarized as follows:

1. By maintaining the same number of sample points, down-sampling extends the temporal range of the input signal, enhancing the network’s ability to extract features efficiently.
2. This paper utilizes current signals instead of vibration signals, reducing sensor costs while maintaining high diagnostic accuracy.
3. The proposed DSC architecture achieves significantly lower memory usage and fewer floating-point operations (FLOPs) compared to traditional CNNs, with negligible impact on accuracy.
4. The application of DoReFa-Net for weight and activation function quantization further reduces memory consumption and computational complexity.

The structure of this paper is as follows: Section 2 provides a comprehensive review of related research on bearing fault diagnosis. Section 3 introduces the proposed DSC architecture in detail, followed by Section 4, which explains its hardware implementation. Experimental results are presented in Section 5, and Section 6 provides the conclusion.

## 2. Related Work

Recent studies have made significant progress in applying machine learning techniques to fault diagnosis for rolling bearings, yielding promising results. These methods generally follow a two-stage process: feature extraction and fault classification, with the success of diagnosis heavily dependent on the quality of feature extraction. Traditional approaches, however, face key challenges, as they often rely on manual feature extraction followed by the use of classifiers for fault categorization. This dependence not only reduces the accuracy of feature detection but also increases computational and implementation costs.

Ref. [19] introduced a fault classification algorithm based on multi-domain feature optimization, leveraging statistical analysis, fast Fourier transform (FFT), and variational mode decomposition (VMD) to extract and optimize fault-relevant features across multiple domains. This approach facilitates the efficient identification of features that are both meaningful and sensitive to fault conditions. Similarly, Ref. [11] presented a feature distance stacking autoencoder (FD-SAE) for rolling bearing fault diagnosis. Utilizing the clear distinctions between normal and faulty data in the CWRU dataset (a widely used benchmark for bearing fault diagnosis), support vector machine (SVM) and FD-SAE methods are applied independently to diagnose normal and faulty data, significantly reducing computational complexity.

Long short-term memory (LSTM) networks, an extension of recurrent neural networks (RNNs) designed to address long-term dependency challenges, have gained significant attention in recent applications. By employing gating mechanisms, LSTMs effectively regulate memory retention, determining how prior states, stored information, and input data are combined. In Ref. [12], the parameters of an LSTM-based classification model were fine-tuned, and L1 regularization was applied to enhance generalization by promoting sparsity and mitigating overfitting.

Ref. [20] proposed a method for bearing signal analysis that integrates variational mode decomposition with hierarchical fuzzy entropy to effectively capture critical signal features. To further reduce human intervention and processing time, AlexNet is utilized for automated feature extraction. Compared to traditional CNNs, AlexNet demonstrates superior diagnostic accuracy by efficiently isolating key information from bearing signals while minimizing the effects of noise and other interference factors.

Given the importance of critical decision-making in machine state monitoring, the outputs of autonomous monitoring systems must prioritize both reliability and interpretability. Ref. [13] introduced a specialized CNN architecture, DecouplEd Feature-Temporal CNN (DEFT-CNN), which integrates analyses across frequency, time, and time–frequency domains to classify fault types in the PU dataset. Using gradient-weighted class activation mapping (Grad-CAM), DEFT-CNN generates interpretable visualizations of key features and temporal dynamics, effectively balancing high diagnostic accuracy with enhanced interpretability.

Ref. [14] introduced a CGAN-2-D-CNN fusion model, which integrates a conditional generative adversarial network (CGAN) with a two-dimensional CNN to address the challenge of bearing fault diagnosis with limited sample sizes. Experimental results indicate that the CGAN-2-D-CNN achieves accuracy comparable to a standalone 2-D CNN when trained on the same original dataset. However, as sample sizes decrease further, the GAN-generated data may exhibit excessive similarity to the original samples, potentially restricting the model’s capacity for generalization.

Ref. [15] proposed an integrated ResNet and GoogLeNet framework for predicting the remaining useful life (RUL) of bearings. To simplify the model and address overfitting, increased stride lengths are employed instead of pooling layers, thereby also reducing computational overhead. GoogLeNet is utilized for feature extraction, leveraging its architecture to capture richer and more detailed data representations. Furthermore, an attention mechanism is incorporated to enhance network convergence efficiency and improve the precision of RUL predictions.

Several studies have focused on addressing resource constraints by developing bearing fault diagnosis methods tailored for embedded systems. For example, Bertocco [21] introduced a machine learning (ML) algorithm optimized for microprocessors to detect early-stage roller bearing faults, including defects in the bearing balls, inner raceways, and outer raceways. This algorithm supports integration into IoT-based predictive maintenance systems via distributed sensor networks, enabling proactive machine failure prevention. Similarly, Ding [22] proposed S-AlexNet, an edge intelligence fault diagnosis approach based on a lightweight convolutional neural network (CNN) using parameter transplantation. Designed for real-time condition monitoring on low-cost embedded systems, S-AlexNet offers a practical solution for edge computing environments. However, both methods rely on vibration signal data, necessitating additional sensors to be installed on the bearing equipment.

In addition, several prior studies have utilized the same dataset as this work, employing current data to classify bearing faults. For instance, Ref. [23] proposed a two-dimensional convolutional neural network (2-D CNN) for feature extraction, emphasizing fault diagnosis through the analysis of both current and vibration signals. Although vibration signals are often preferred due to their spectral characteristics, which are easier to extract compared to current signals, their dependence on vibration sensors significantly increases costs, posing challenges for practical applications. The classification in Ref. [23] encompasses four fault conditions—inner race fault, outer race fault, combined inner and outer race fault, and normal. In contrast, this study focuses on three fault conditions with a particular emphasis on computational efficiency.

Furthermore, Ref. [24] achieved superior accuracy by leveraging synthetic current data. The approach employs multiple convolutional and pooling operations to extract intrinsic features from raw mechanical signals, which are subsequently classified into mechanical health states through fully connected layers. Additionally, Ref. [25] introduced an innovative transfer learning framework for bearing fault diagnosis, utilizing a convolutional neural network (CNN) to transfer knowledge from artificial damage data to real-world damage scenarios. The CNN model is pre-trained on easily obtained artificial damage samples, fine-tuned with a small number of real damage samples, and some layers are frozen to retain shared features between the two datasets.

However, these studies, which utilize the same dataset as this work, seldom address parameter efficiency or computational complexity. Furthermore, they predominantly rely on software-based architectures that transmit sensor data to a central server for processing, limiting their ability to satisfy real-time operational requirements. In practical industrial environments, real-time decision-making is often critical. Consequently, this paper aims to develop a hardware accelerator that optimizes both resource efficiency and computational complexity, delivering high accuracy while maintaining real-time performance.

In recent developments addressing hardware deployment, Chung [26] proposed a one-dimensional convolutional neural network (CNN) hardware accelerator for real-time bearing condition monitoring. Their approach incorporates techniques such as down-sampling and quantization to enhance the efficiency of neural network models. Nevertheless, the adoption of quaternary quantization, aimed at substantial memory reduction and real-time fault diagnosis in embedded systems, remains an active area of investigation. Despite these contributions, the parameter count in their model could be further optimized.

Despite their strong performance in various fault diagnosis applications, CNN-based classification models face significant challenges. First, deeper networks require extensive parameter sets, resulting in substantial resource demands for deployment and training, which hinders efficient hardware implementation. Moreover, industrial applications necessitate fault diagnosis models that are both memory-efficient and capable of high-speed predictions, posing challenges for direct deployment on production lines. Second, most diagnostic models assume that the training and test sets share the same data distribution, which overlooks the model’s ability to generalize to new scenarios or datasets.

Consequently, variations in operating conditions or environments can markedly degrade the model’s performance, even for tasks that are similar to the original.

## 3. The Proposed DSC Model Architecture

### 3.1. Bearing Data Preprocessing

To provide a foundation for the proposed methodology, the dataset employed in this study is first described. The Paderborn University-bearing dataset is used to analyze key features related to bearing conditions. This dataset includes four categories of bearing fault data, with each category containing a distinct number of data points. Table 1 summarizes the proportion of each fault type.

**Figure 1:**
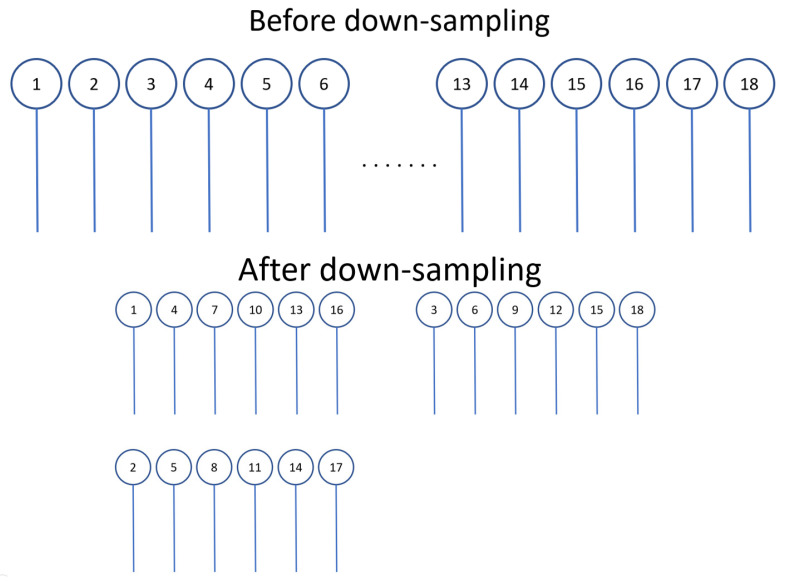
The operation of down-sampling, while the down-sampling factor set to 3.

During the data-preprocessing stage, down-sampling is applied to the dataset to reduce redundancy and improve processing efficiency. Figure 1 illustrates the process of down-sampling in a pipeline, where the original signal is sampled at specified intervals based on a down-sampling factor. For example, with a factor of 1, the raw data are retained without modification, while a factor of 3 skips two data points between each sample. To maintain the overall data volume, the skipped data points are repurposed to generate additional training samples, ensuring that the total number of training images remains unchanged.

Experimental accuracy results from Ref. [26] indicate that a down-sampling factor of 10 achieves the best performance. Consequently, this study adopts the same factor. However, this choice necessitates observing the data over a longer time window to ensure sufficient information is captured for effective analysis.

Down-sampling methods are notable for their ability to reduce noise, making them particularly suitable for real-time processing. Additionally, down-sampling allows the model to associate each data point with an extended time interval, thereby retaining sufficient information for accurate feature extraction. To avoid potential data leakage caused by splitting adjacent data points between the training and test sets, the dataset is first partitioned into 80% for training and 20% for testing, after which down-sampling is applied separately to each subset. Table 2 presents the amount of training and test data for each label.

### 3.2. Depthwise Separable Convolution Architecture and DoReFa-Net Quantization

Adjusting the network architecture requires specifying several key hyperparameters before training, including kernel size, the number of convolutional layers, the learning rate, and the number of epochs. The input data are encoded as fixed-point numbers, whereas the weights are stored as floating-point numbers to balance computational efficiency and precision. While increasing the number of convolutional layers generally improves accuracy, excessive layers or epochs can lead to overfitting, where the model achieves high accuracy on the training data but performs poorly on test data. Therefore, identifying an optimal network architecture requires iterative training and careful evaluation of performance metrics.

**Figure 2:**
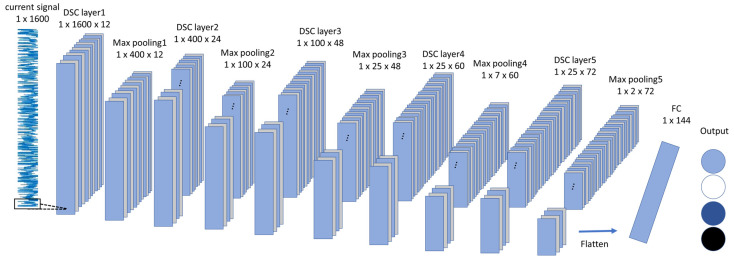
The overview architecture of the proposed 1-D DSC architecture.

This paper proposes a bearing fault diagnosis method leveraging current signals, utilizing the one-dimensional (1-D) DSC architecture illustrated in Figure 2. The model processes 1600 data points per input image and incorporates five DSC layers, which consist of six pointwise convolutional layers and five depthwise convolutional layers. This architecture is designed to efficiently extract features while minimizing computational complexity.

**Figure 3:**
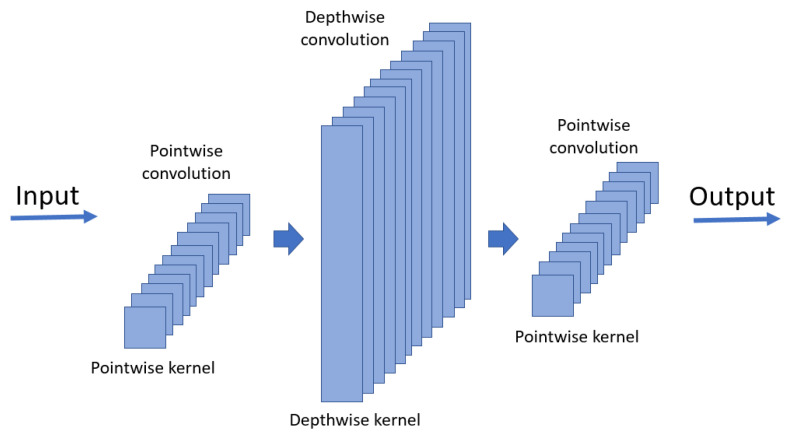
The operation of DSC in the first layer.

Table 3 shows the accuracy of the proposed DSC architecture under varying numbers of output channels. Testing various architectural configurations revealed that standard DSCs often fail to achieve satisfactory accuracy. This limitation arises from the structure of DSC architectures, which combine depthwise and pointwise convolutions. Depthwise convolutions are constrained by a configuration of 1 × kernel size × input channels configuration. Since the input to the first layer is typically set to 1, its depth is dependent on the preceding layer. To address this issue, as illustrated in Figure 3, a pointwise convolution layer is introduced before the first depthwise convolution layer. This modification enables the first layer to independently increase in depth regardless of the input image dimensions. Experimental results demonstrate that incorporating a pointwise convolution layer in the first DSC layer significantly enhances the accuracy of the proposed model. In this study, the kernel size was set to 7, and the model was trained for 500 epochs.

In this paper, each convolution operation in the DSC layer is directly followed by batch normalization and ReLU activation. Max-pooling is applied after each DSC layer, and a fully connected (FC) layer serves as the final layer in the network. The hyperparameters for model training were configured as follows: 400 training epochs, 400 retraining epochs, a batch size of 128, and a learning rate of 10^−3^. The model architecture was established through iterative experimentation employing a Train-Test Split strategy to partition the dataset into training and testing subsets. During these iterations, the architecture was refined through multiple adjustments. Once finalized, the model underwent cross-validation using various training and test data sets to evaluate its stability and robustness.

Neural network applications deployed on edge devices are constrained by limited computational resources, including storage, memory, power consumption, and latency. As shown in Table 3, the proposed architecture comprises a total of 11 K parameters, of which convolution operations contribute 10 K, accounting for 87% of the total. Consequently, reducing the precision of parameters in convolution operations is a crucial step to significantly lower memory usage and computational overhead.

Neural network computations rely on numerous parameters and inputs, which are typically stored in the 32-bit floating-point format (float32). Quantization reduces memory storage requirements and computational costs by converting float32 data into lower-precision formats, such as 8-bit or 16-bit representations. This process enables more efficient computation while potentially balancing trade-offs in model accuracy.

Quantization techniques are broadly categorized into post-training quantization and quantization-aware training. Post-training quantization employs tools to quantize a pre-trained model, optimizing it for efficient deployment. Specifically, the checkpoint file (.ckpt) generated after training in TensorFlow is loaded, and quantization is applied to both weights and activations. Post-training quantization can be further divided into two approaches: weight-only quantization, where only the weights are quantized, and full quantization, which involves quantizing both weights and activations. The latter approach requires additional computation to determine the dynamic range of activation outputs, ensuring proper scaling during inference. In this paper, we adopt post-training quantization of both weights and activations to achieve efficient deployment while maintaining computational and memory efficiency.

Neural network computations often involve numerous parameters, a significant portion of which may carry redundant or non-essential information. Quantization, an effective technique for optimizing these models, helps reduce memory usage and hardware resource demands. In this work, the proposed DSC architecture employs DoReFa-Net quantization to minimize parameter bit-width. DoReFa-Net [27] simplifies backpropagation by calculating a single scaling factor for the total output of each convolutional layer, rather than for each convolutional kernel output individually. To enhance comprehension of the quantization methodology utilized in this work, the subsequent sections introduce the quantization approach proposed in DoReFa-Net [27].

The straight-through estimator (STE) is used to address the zero-gradient problem. STE can be seen as an operator that permits flexible forward and backward operations. Its forward and backward passes are defined in Equations (1) and (2), where ${quantize}_{k}\left(x\right)$ quantizes the input to k bits. In cases of zero gradients, the derivative is zero during backpropagation, preventing the network from learning and updating weights. To avoid this, STE approximates the gradient by setting the input gradient equal to the output gradient via a threshold function, thereby circumventing the zero-gradient issue.

(1) $$\mathrm{Forward}:\;{quantize}_{k}\left(x\right)=\;\frac{1}{2^{k}-1}\;round((2^{k}-1)x)$$

(2) $$\mathrm{Backward}:\;\frac{\partial c}{\partial r_{i}}=\frac{\partial c}{\partial r_{O}}$$

To mitigate the significant quantization error caused by a large input data range, the tanh function is applied to constrain the weights within the range of [−1, 1]. Following this step, the weights are quantized to k bits, as described in Equation (3):

(3) $$f_{w}^{k}(r)=2{quantize}_{k}(\frac{tanh(r)}{2max(|tanh(r)|)}+\frac{1}{2})-1$$

Table 4 presents the test accuracy of the proposed DSC architecture with and without retraining, supporting the adoption of retraining in this paper’s quantization method. Additionally, Table 5 shows test accuracy across different weight bit-widths. Reducing bit-width from 32 to 8 bits resulted in only a 0.45% accuracy loss, highlighting the effectiveness of weight quantization in preserving accuracy while improving memory efficiency.

In this study, DoReFa-Net [27] quantization reduces the weight precision from 32 bits to 8 bits, thereby lowering the weight storage requirement by 75%. Overall, the combined application of quantization and parameter optimization accelerates inference in the proposed DSC architecture, enhancing its suitability for hardware implementation in resource-constrained conditions.

In DoReFa-Net quantization, experimental results indicate that a bounded activation function is essential to scale input activations to a specific range, ensuring stable model convergence. If input activations are not scaled, the model may struggle to converge accurately. In this paper, the scaling factor is selected based on the observed range of activation values, with a denominator that is a power of two (e.g., 2, 4, and 8) to effectively limit the range and minimize quantization errors. This approach reduces computational complexity by enabling multiplication operations to be performed using bit shifting and addition instead of multipliers. Specifically, Equation (4) defines the bounded activation function used in this paper, and a scaling factor of 0.078125 (i.e., 5/64) is selected to constrain the activation function, as shown in Equation (5):

(4) h(x)=clip(x,0,1)

(5) h(x)=clip(x×0.078125,0,1)

Table 6 shows the test accuracy for different activation bit-widths in DoReFa-Net quantization. Experimental results indicate that test accuracy is sensitive to activation bit-width, dropping by approximately 2% as bit-width decreases from 10 to 6. Thus, the activation bit-width is set to 8 bits, yielding an accuracy of 97.52%.

Table 7 shows simulation data used to determine the bit-width for the weight lookup table. With the weight bit-width established, the total memory required to store the weights can be calculated based on the number of parameters, yielding a total storage requirement of 154,980 bits.

Since Batch Normalization (BN) parameters cannot be quantized via retraining, a fixed-point method is used to reduce their bit-width. Although BN operations require only 768 parameters, reducing bit-width is still advantageous for hardware implementation. BN involves four parameters: gamma, beta, mean, and variance. As shown in Equations (6)–(8), these four parameters can be combined into two, reducing the parameter count. Here, $\mu_{B}$ represents the mean, and $\alpha_{B}^{2}$ represents the variance.

(6) $$\gamma^{\prime }=\frac{1}{\sqrt{\alpha_{B}^{2}}}\gamma$$

(7) $$\beta^{\prime }=-(\mu_{B}\gamma^{\prime })+\beta$$

(8) $$y_{i}=\gamma^{\prime }x_{i}+\beta^{\prime }$$

Table 8 presents the accuracy results for BN parameters across different bit-widths. The integer bit-width is set to 8, based on the observed maximum and minimum values of γ and β, resulting in a representable range of −128 to 127. For fractional bits, it was observed that accuracy drops significantly when the fractional bit count is less than 8, while an 8-bit fractional width allows accuracy to be maintained at 97%. Consequently, the total bit-width for BN parameters can be reduced from a 32-bit floating point to a 16-bit fixed-point, composed of 8 integer bits and 8 fractional bits, thereby reducing parameter bit-width by 50%. Given the number of BN parameters, the total bit requirement for BN parameters can be calculated as 12,288 bits.

The results of testing different activation bit-widths are presented in Table 9, with the integer bit-width set to 1, corresponding to a representable range of 0 to 1. A noticeable drop in accuracy was observed between total bit-widths of 12 and 13. Consequently, the optimal bit-width for activation after fixed-point quantization is set to 13.

## 4. Hardware Implementation

**Figure 4:**
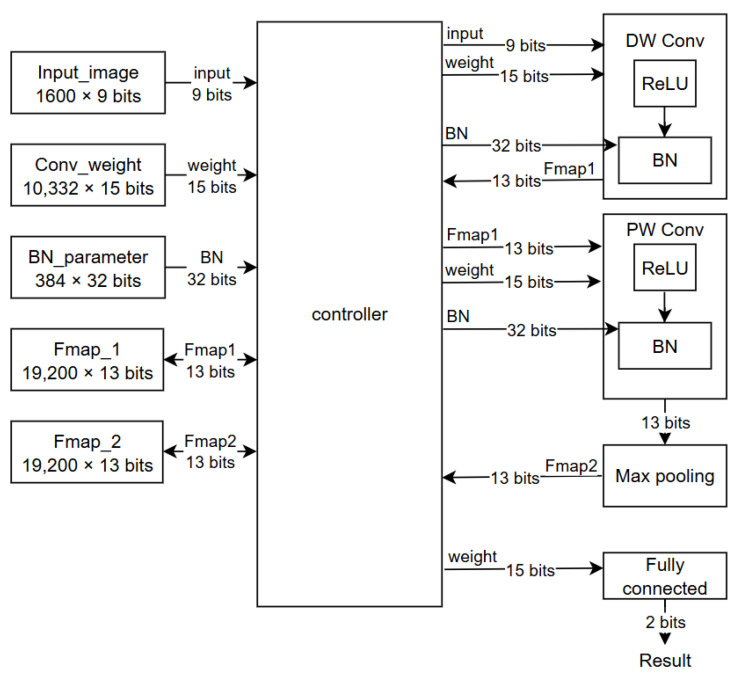
Overall architecture of the proposed DSC hardware design.

Figure 4 illustrates the hardware architecture of the proposed DSC. Before computation begins, the Input_image stores a preprocessed 1 × 1600 input image. The controller then applies zero padding to the input image. During each clock cycle, the controller reads weight values stored in Conv_weight and retrieves 15-bit actual weight values from the lookup tables for each layer. Weights are quantized using DoReFa-Net quantization, limiting each layer to a maximum of 256 weight values.

The controller transmits the actual weight values and input image to the DW Conv block, where the DW Conv module performs 1-D depthwise convolution, ReLU, and batch normalization. Given a kernel size of 7, the module applies multiply-accumulate (MAC) operations to the input and weights. In the ReLU block, if the most significant bit (MSB) is 1, indicating a negative value, the ReLU output is set to 0; otherwise, it outputs the accumulated result. The output from the ReLU block is then sent to the BN block. Before batch normalization, the controller retrieves 16-bit γ and β values from the BN_parameter block, using these to scale and shift the ReLU output by multiplying it by γ and adding β. The output of the BN operation is then quantized to 13 bits and stored in Fmap_1.

The PW_Conv block then reads weights from the Conv_weight block and feature maps from Fmap_1, generated by the DW_Conv block, using the same approach as the DW_Conv block. The PW_Conv block performs ReLU and BN operations, requiring parameters from the BN_parameter block. The output of the PW_Conv block is passed to the Max Pooling block, where, after four values are transferred, the max pooling operation is performed. The result from the Max Pooling block is stored in Fmap_2 (one of the ping-pong RAM blocks). The remaining operations follow the same processing steps as in the DW_Conv and PW_Conv blocks. Using a ping-pong architecture avoids memory conflicts between the memory requirements of the previous layer and the output storage of the current layer, while also reducing overall memory usage.

As shown in Table 10, quantization is a crucial process for reducing memory resource usage in neural networks. After quantization, the bit usage for convolution weights is reduced by approximately 53.1%, BN parameters by 50%, and feature map memory usage by 59.4%, resulting in an overall reduction in total bit requirements of 57.9%.

In the proposed DSC hardware design, a single-port read-only memory (ROM) is employed to store the model parameters, as the circuit is synthesized and implemented on an FPGA (Field Programmable Gate Array). The single-port ROM IP is generated using the IP block generator available in AMD Vivado 2020.2 Design Suite software. Table 11 details the composition and total bit usage of each storage block. The cumulative storage requirement for this circuit implementation is 774 K bits. However, due to a minimum memory block size constraint of 18 K bits, the total memory allocation for the DSC hardware circuit was adjusted, resulting in a memory usage of 420 K bits.

## 5. Experimental Results

**Figure 5:**
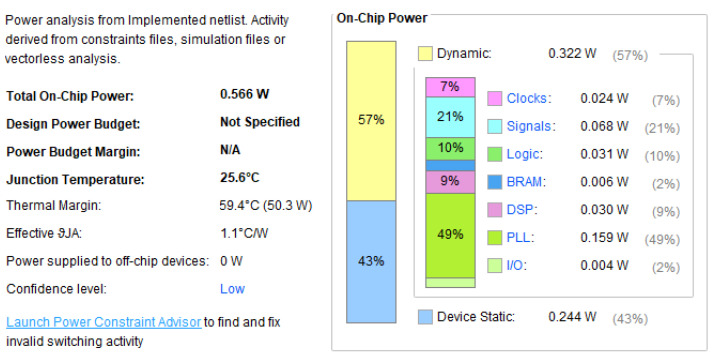
The power analysis of the proposed DSC hardware design at 50 MHz.

The power analysis for the FPGA implementation is shown in Figure 5. During testing, the clock frequency is set to 50 MHz, resulting in a total power consumption of 566 mW at a temperature of 25.6 °C. Of the total power, 57% is attributed to dynamic power, while 43% is attributed to static power. Here, BRAM refers to the built-in memory of the FPGA, meaning that smaller BRAM usage translates to lower resource consumption.

The test data are stored in the FPGA’s memory blocks to verify circuit functionality. Multiple entries of test data can be stored within these blocks. However, Vivado imposes a memory block size limit, allowing only 655 images to be stored at once when the test data size is 1 × 1600. As a result, each test on the FPGA is restricted to 655 images, requiring successive cycles of different test data to fully verify the functionality of the proposed DSC architecture.

Table 12 presents the relative accuracy of the proposed DSC architecture across each implementation stage. From TensorFlow to FPGA deployment, the total accuracy loss is approximately 0.47%.

The experimental results of various studies using the same dataset are compared in Table 13. The model proposed in Ref. [23] employed a two-dimensional (2-D) CNN for feature extraction. The results are evaluated using actual damage observed in current and vibration signals, as the vibration signal’s spectral characteristics are easier to extract than those of the current signal. This makes vibration signals more effective for fault diagnosis. However, the sensors needed for collecting vibration signals are costly, raising the overall expense of fault diagnosis. The classification labels used are inner race fault, outer race fault, combined inner and outer race fault, and normal. In contrast, Ref. [23] classified only three fault conditions.

Ref. [24] used artificial current data, achieving higher accuracy than this work but requiring at least 70 times more parameters. Moreover, relying on synthetic rather than actual damage data may lead to less reliable judgments in practical applications. Refs. [3,25] used mixed data from current and vibration signals and classified only three fault labels. However, both models have higher parameter counts and FLOPs than this work, making this model more suitable for efficient hardware implementation.

More recently, ref. [26] adopted a one-dimensional CNN architecture with a new quaternary quantization method to improve accuracy compared to ternary quantization. Although the proposed method is suitable for hardware implementation and achieves real-time performance, its architecture still results in a significantly higher number of parameters compared to the proposed method.

**Figure 6:**
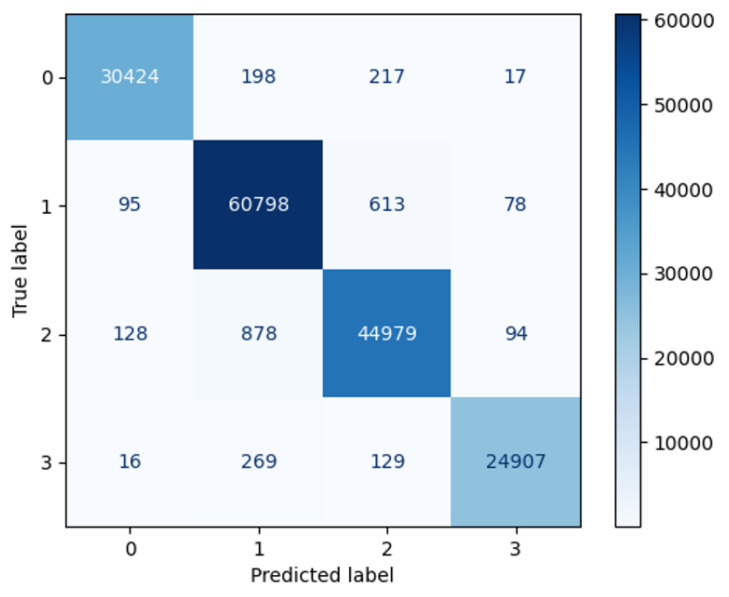
The confusion matrix of the software training result.

To provide a clearer representation of the software-based results, Figure 6 presents the confusion matrix for the software training outcomes. In this figure, the term ‘label’ refers to the classification type specific to this study (as detailed in Table 1). The ‘True label’ represents the actual type of the sample, while the ‘Predicted label’ indicates the type assigned by the model. This confusion matrix demonstrates that the model achieves a consistent level of accuracy across various label categories. Additionally, Table 14 provides detailed metrics, including precision, recall, and F1-score for each type. As shown in Table 14, the F1-scores for all labels exceed 97%, indicating that the training results effectively balance precision and recall, with no significant bias toward any specific type.

The following sections examine whether the proposed DSC hardware design can perform real-time bearing failure analysis. Table 15 lists the clock cycles required for each layer’s computation. According to the PU dataset specifications, the current signal data has a sampling rate of 64 K samples per second. With a down-sampling factor of 10, this rate is reduced to 6.4 K samples per second, meaning each data point is received every 156,250 ns. Since the initial computation stage requires 1600 data points to begin processing, subsequent calculations cannot proceed until this initial data collection is complete. Consequently, the waiting time for the first image is 250,000,000 ns, as shown in Equation (9).

(9) 250,000,000 ns=156,250 ns × 1600

As the proposed DSC hardware design receives the first image, new data points continue to be input during ongoing calculations. However, memory resources cannot be freed until the current computation is completed. Therefore, an additional buffer is needed to temporarily store incoming sensor data until the previous image’s processing is finished. In this paper, the maximum clock frequency is set to 50 MHz. Thus, 12.5 million cycles are required to gather the 1600 data points, and the DSC model calculation requires 525,889 cycles. Consequently, approximately 67 data points need to be stored in additional buffers to accommodate the calculation delay, as shown in Equation (10):

(10) 525,889 × 20 ns ÷ 156,250 ns ≈ 67

Taking into account both the image acquisition time and the diagnostic processing time of the DSC hardware design, the total response time for the proposed DSC hardware design is 0.26 s, as calculated in Equation (11):

(11) 0.26 s=250,000,000 ns+525,889 cycles×20 ns

## 6. Conclusions

This paper utilizes bearing current signals from the Paderborn University dataset and proposes a DSC network architecture designed to reduce parameter count and computational load. Additionally, it incorporates the DoReFa-Net quantization method to decrease the bit-width of model parameters. Testing results demonstrate that the proposed DSC achieves significant resource savings with minimal accuracy loss.

The proposed DSC hardware design is initially developed using hardware description language (HDL) and verified through circuit simulation before being implemented on the AMD Virtex-7 FPGA VC707 evaluation board. This setup enables the final accuracy evaluation of the DSC hardware implementation and resource analysis. Additionally, the paper examines the requirements for real-time bearing fault diagnosis, concluding that the DSC hardware design needs only an additional buffer to store 67 data points.

The accuracy of the proposed DSC hardware design implemented on the FPGA is ultimately 96.12%, with only a 0.47% reduction in accuracy compared to that achieved using TensorFlow.

To sum up, this paper presented a hardware implementation for real-time bearing fault detection, focusing on reducing hardware costs while maintaining acceptable accuracy. In the future, authors will refer to some excellent previous works [28,29,30] to consider exploring techniques such as early exit mechanisms and model modularization to enhance flexibility and further reduce costs. Additionally, incorporating diverse real-world datasets will improve the model’s adaptability. In our opinion, the key limitation of studies in this field is that real-world scenarios often differ from existing datasets. While high accuracy was achieved by using real-world data, models trained solely on standard datasets may struggle in real-world applications, highlighting the need for improvements in model generalization.

## Figures and Tables

**Table 1:** Network setting label and the corresponding fault type.

Label	Fault Type	Data Amount
0	Healthy	6
1	Outer race fault (OR)	12
2	Inner race fault (IR)	9
3	Combine outer and inner race fault (CR)	5

**Table 2:** The amount of training and test data in each label.

Label	Amount of Training Data	Amount of Test Data
0	115,260	28,815
1	230,860	57,715
2	173,280	43,320
3	96,160	24,040
Total data	615,560	153,890

**Table 3:** The accuracy in different conditions with proposed DSC.

Version	Output Channels in Each Layer					Param #	Accuracy
	C1	C2	C3	C4	C5		
1	12	18	48	60	72	9.9 K	97.71%
2	12	12	48	60	72	9.5 K	95.96%
3	8	24	48	60	72	10 K	96.54%
4	8	36	48	60	72	10 K	96.67%
5	8	12	48	60	72	9.4 K	95.57%
6	12	24	48	60	72	11 K	98.27%

**Table 4:** The test accuracy with retraining and without retraining.

Result	Quantization Method	Retrain	Testing Accuracy
1	DoReFa-Net	No	94.47%
2	DoReFa-Net	Yes	98.27%

**Table 5:** The test accuracy of different weight bits.

Result	Bit-Width of Weight	Testing Accuracy
1	32	98.27%
2	10	98.11%
3	9	97.98%
4	8	97.82%
5	7	97.15%
6	6	97.09%
7	5	91.64%

**Table 6:** The test accuracy with different bit-width of activation in DoReFa-Net.

Result	Bit-Width of Activation	Testing Accuracy
1	32	98.27%
2	10	97.73%
3	8	97.52%
4	7	97.04%
5	6	95.74%

**Table 7:** The lookup table for determining the bit-width of weight.

The Lookup Table for Bit-Width of Weight			
Integer Bits	Decimal Bits	Total Bits	Test Accuracy
2	13	15	97.17%
2	12	14	96.93%
2	11	13	96.45%
2	10	12	96.19%
2	9	11	95.19%
2	8	10	89.47%

**Table 8:** The table for determining the bit-width of BN parameters.

Bit-Width of BN Parameters			
Integer Bits	Decimal Bits	Total Bits	Test Accuracy
8	15	23	97.15%
8	13	21	97.12%
8	11	19	97.12%
8	9	17	97.03%
8	8	16	97.03%
8	7	15	96.12%
8	6	14	93.28%

**Table 9:** The table for determining the lookup table bit-width of activation fixed point.

The Lookup Table Bit-Width of Activation			
Integer Bits	Decimal Bits	Total Bits	Test Accuracy
1	15	16	96.99%
1	14	15	96.95%
1	13	14	96.84%
1	12	13	96.59%
1	11	12	95.75%
1	10	11	94.31%

**Table 10:** The comparison of memory usage after fixed-point parameter.

Memory Type	Memory Name	Total Bits Before the Fixed Point	Total Bits After the Fixed Point	Reduction Ratio
ROM	Conv_Weight	330,624	154,980	53.125%
	BN_parameter	24,576	12,288	50%
RAM	Fmap_1	614,400	249,600	59.375%
	Fmap_2	614,400	249,600	59.375%
Sum of all bits		1,584,000	666,468	**57.925%**

**Table 11:** Size and composition of each memory block.

Memory Name	18 Kbits RAM	36 Kbits RAM	Total Kbits
Input_image	1	0	18
Conv_weight	3	4	198
BN_parameter	1	0	18
Fmap_1	5	5	270
Fmap_2	5	5	270
Total	15	14	774

**Table 12:** The test accuracy at each implementation stage.

Each Stage Operation	Accuracy
Input data fixed-point training (9 bits)	97.52%
Weight value fixed-point (15 bits)	97.17%
BN parameters fixed-point (16 bits)	97.03%
Feature map fixed-point (13 bits)	96.59%
FPGA implement	96.12%

**Table 13:** Comparison table at the software level.

	[23] IEEE TIM ‘20		[24] IEEE Access ‘20	[25] ICICAS ‘19	[3] IEEE TIM ‘21	[26] Sensors ’23	Proposed Work	
Dataset	PU		PU	PU	PU	PU	PU	
Type of damage	Real damage		Artificial damage	Mixed damage	Mixed damage	Mixed damage	Mixed damage	
Signal type	Vibration	Current	Current	Current	Vibration	Current	Vibration	Current
Architecture	2-D CNN		1-D CNN	1-D CNN	1-D CNN	1-D CNN	1-D DSC	
Data pre-processing	Gray image		N/A	N/A	N/A	Down-sampling and fixed-point	Down-sampling and fixed-point	
Image size	80 × 80		1 × 1200	1 × 1800	1 × 696	1 × 1600	1 × 1600	
Number of parameters	25,810		>750,000 ^1^	40,448	33,280	75,436	11,868	
FLOPs	16,781,497		N/A	16,767,933	7,418,436	N/A	1,203,128	
Type of classification	Fault location (3 types without IR + OR combined fault)		Fault location (3 types without IR + OR combined fault)	Fault location (4 types)	Fault location (3 types without IR + OR combined fault)	Fault location (4 types)	Fault location (4 types)	
Accuracy	99.4%	98.3%	99.36%	97.78%	98.16%	98.58%	99.45%	98.27%

^1^: Only count the parameters of the fully connected layer.

**Table 14:** Precision, recall, and F1-score for each label.

Label	Precision	Recall	F1-Score
Label 0	99.22%	98.60%	98.91%
Label 1	97.84%	98.72%	98.28%
Label 2	97.91%	97.61%	97.76%
Label 3	99.25%	98.36%	98.80%

**Table 15:** Cycle usage in each layer calculation.

	# Cycles
Layer 1	19,219
Layer 2	19,405
Layer 3	19,265
Layer 4	5004
Layer 5	182,690
Layer 6	2808
Layer 7	149,954
Layer 8	2016
Layer 9	85,382
Layer 10	1440
Layer 11 + FC	38,676
Total	525,889

## Data Availability

Data are contained within the article.
